# Current Awareness on Comparative and Functional Genomics

**DOI:** 10.1002/cfg.55

**Published:** 2001-02


**Comparative and Functional Genomics**
*Comp. Funct. Genom.* 2001; **2:** 49–56

### Current awareness on comparative and functional genomics

### 1 Reviews & symposia

1999. Special issue: Proceedings of the Ist Annual Georgia Genetics Symposium, held October 8 - 10, 1999 - University of Georgia, Athens, Ga, USA (Contains many papers on genome evolution). *Genetica* **107:** (1-3)

2000. Special issue: AGA symposium issue - Genome Diversity and Evolution, the Pennsylvania State University, University Park, Pa, USA, June 12-13 1999. *J Hered* **91:** (3)

2000. Special issue: Biotic interactions - Genomics and co-evolution. *Curr Opin Plant Biol* **3:** (4)

2000. Special issue: Chemical mutagenesis in mice. *Mamm Genome* **11:** (7)

2000. Special issue: Pharmaceutical proteomics. *Electrophoresis* **21:** (11)

Andersson JO, Andersson SGE*. 2000. *Uppsala Univ, Dept Mol Evolut, Evolutionary Biol Ctr, Norbyvagen 18C, SE-75236 Uppsala, Sweden. A century of typhus, lice and rickettsia. *Res Microbiol* **151:** (2) 143.

Andrade MA, Bork P*. 2000. *European Mol Biol Lab, Meyerhofstr 1, DE-69012 Heidelberg, Germany. Automated extraction of information in molecular biology (Minireview). *FEBS Lett* **476:** (1-2) 12.

Bell JD, Taylor-Robinson SD. 2000. Hammersmith Hosp, Imperial Coll, Sch Med, Robert Steiner MRI Unit, MRC, Clin Sci Ctr, Du Cane Rd, London W12 0HS, England. Assessing gene expression *in vivo*: Magnetic resonance imaging and spectroscopy (Review). *Gene Ther* **7:** (15) 1259.

Benner SA, Chamberlin SG, Liberles DA, Govindarajan S, Knecht L. 2000. Univ Florida, Dept Chem, Gainesville, Fl 32611, USA. Functional inferences from reconstructed evolutionary biology involving rectified databases: An evolutionarily grounded approach to functional genomics. *Res Microbiol* **151:** (2) 97.

Broder S, Venter JC. 2000. Celera Genomics, Rockville, Md 20850, USA. Sequencing the entire genomes of free-living organisms: The foundation of pharmacology in the new millenium. *Annu Rev Pharmacol Toxicol* **40:** 97.

Brosch R, Gordon SV, Eiglmeier K, Garnier T, Cole ST*. 2000. *Inst Pasteur, Unite Genet Mol Bacterienne, 28 rue Docteur Roux, FR-75724 Paris 15, France. Comparative genomics of the leprosy and tubercle bacilli. *Res Microbiol* **151:** (2) 135.

Brosch R, Gordon SV, Pym A, Eiglemeier K, Garnier T, Cole ST. 2000. Address as above. Comparative genomics of the mycobacteria (Review). *Int J Med Microbiol* **290:** (2) 143.

Brunder W, Karch H. 2000. Univ Wurzburg, Inst Hyg & Mikrobiol, Josef Schneider Str 2, DE-97080 Wurzburg, Germany. Genome plasticity in Enterobacteriaceae (Review). *Int J Med Microbiol* **290:** (2) 153.

Brune W, Messerle M, Koszinowski UH. 2000. Univ Munich, Max von Pettenkofer Inst, Dept Virol, DE-80539 Munich, Germany. Forward with BACs: New tools for herpesvirus genomics (Review). *Trends Genet* **16:** (6) 254.

Carroll SB. 2000. Univ Wisconsin, Howard Hughes Med Inst, RM Bock Labs, 1525 Linden Dr, Madison, Wi 53706, USA. Endless forms: The evolution of gene regulation and morphological diversity (Minireview). *Cell* **101:** (6) 577.

Chakraborty T, Hain T, Domann E. 2000. Justus Liebig Univ Giessen, Inst Med Mikrobiol, Frankfurter Str 107, DE-35392 Giessen, Germany. Genome organization and the evolution of the virulence gene locus in *Listeria* species (Review). *Int J Med Microbiol* **290:** (2) 167.

Coelho PSR, Kumar A, Snyder M. 2000. Yale Univ, Dept Mol Cellular & Dev Biol, POB 208103, New Haven, Ct 06520, USA. Genome-wide mutant collections: Toolboxes for functional genomics. *Curr Opin Microbiol* **3:** (3) 309.

De Angelis DA. 1999. Mem Sloan Kettering Canc Ctr, Cellular Biochem & Biophys Program, 1275 York Ave, Box 251, New York, NY 10021, USA. Why FRET over genomics? (Review). *Physiol Genomics* **1:** (2) 93.

Debouck C, Metcalf B. 2000. SmithKline Beecham Pharmaceut, Discovery Chem & Platform Technol, Res & Dev, King of Prussia, Pa 19406, USA. The impact of genomics on drug discovery. *Annu Rev Pharmacol Toxicol* **40:** 193.

Denison SH. 2000. Eckero Coll, Collegium Nat Sci, St Petersburg, Fl 33711, USA. pH regulation of gene expression in fungi (Review). *Fungal Genet Biol* **29:** (2) 61.

Doolittle RF. 2000. Univ Calif San Diego, Ctr Mol Genet, La Jolla, Ca 92093, USA. Searching for the common ancestor. *Res Microbiol* **151:** (2) 85.

Doolittle WF. 2000. Dalhousie Univ, Canadian Inst Adv Res, Dept Biochem & Mol Biol, Halifax, Nova Scotia, Canada B3H 4H7. The nature of the universal ancestor and the evolution of the proteome. *Curr Opin Struct Biol* **10:** (3) 355.

Eisenberg D, Marcotte EM, Xenarios I, Yeates TO. 2000. UCLA, Inst Mol Biol, Box 951570, Los Angeles, Ca 90095, USA. Protein function in the post-genomic era. *Nature* **405:** (6788) 823.

Emilien G, Ponchon M, Caldas C, Isacson O, Maloteaux JM. 2000. 127 rue Henri Prou, FR-78430 Les Clayes-sous-Bois, France. Impact of genomics on drug discovery and clinical medicine (Review). *Mon J Assoc Physician* **93:** (7) 391.

Galperin MY, Koonin EV*. 2000. *NIH, Natl Lib Med, Natl Ctr Biotechnol Informat, Bethesda, Md 20894, USA. Who's your neighbor? New computational approaches for functional genomics (Review). *Nat Biotechnol* **18:** (6) 609.

Greenfield A. 2000. MRC Mammalian Genet Unit, Harwell OX11 0RD, England. Applications of DNA microarrays to the transcriptional analysis of mammalian genomes (Review). *Mamm Genome* **11:** (8) 609.

Harrington CA, Rosenow C, Retief J. 2000. Affymetrix Inc, 3380 Cent Expressway, Santa Clara, Ca 95051, USA. Monitoring gene expression using DNA microarrays. *Curr Opin Microbiol* **3:** (3) 285.

Hautefort I, Hinton JCD*. 2000. *AFRC, Inst Food Res, Norwich Res Pk, Norwich NR4 7UA, England. Measurement of bacterial gene expression *in vivo*. *Philos Trans R Soc Lond B* **355:** (1397) 601.

Hecker M, Engelmann S. 2000. Univ Greifswald, Inst Mikrobiol, FL Jahn Str 15, DE-17487 Greifswald, Germany. Proteomics, DNA arrays and the analysis of still unknown regulons and unknown proteins of *Bacillus subtilis* and pathogenic Gram-positive bacteria (Review). *Int J Med Microbiol* **290:** (2) 123.

In order to keep subscribers up-to-date with the latest developments in their field, this current awareness service is provided by John Wiley & Sons and contains newly-published material on comparative and functional genomics. Each bibliography is divided into 16 sections. 1 Reviews & symposia; 2 General; 3 Large-scale sequencing and mapping; 4 Genome evolution; 5 Comparative genomics; 6 Pathways, gene families and regulons; 7 Pharmacogenomics; 8 Large-scale mutagenesis programmes; 9 Functional genomics; 10 Transcriptomics; 11 Proteomics; 12 Protein structural genomics; 13 Metabolomics; 14 Genomic approaches to development; 15 Technological advances; 16 Bioinformatics. Within each section, articles are listed in alphabetical order with respect to author. If, in the preceding period, no publications are located relevant to any one of these headings, that section will be omitted.

Copyright © 2001 John Wiley & Sons, Ltd.

Hoheisel JD, Vingron M. 2000. Deutsch Krebsforschungszentrum, Neuenheimer Feld 506, DE-69120 Heidelberg, Germany. Transcriptional profiling: Is it worth the money? *Res Microbiol* **151:** (2) 113.

Huynen M, Snel B, Lathe W, Bork P. 2000. European Mol Biol Lab, Meyerhofstr 1, DE-69117 Heidelberg, Germany. Exploitation of gene context. *Curr Opin Struct Biol* **10:** (3) 366.

Jacobs DI, Van der Heijden R, Verpoote R. 2000. Leiden/Amsterdam Ctr Drug Res, Gorlaeus Labs, Div Pharmacog, PO Box 9502, NL-2300 RA Leiden, The Netherlands. Proteomics in plant biotechnology and secondary metabolism research. *Phytochem Anal* **11:** (5) 277.

Jones DT. 2000. Brunel Univ, Dept Biol Sci, Uxbridge UX8 3PH, England. Protein structure prediction in the postgenomic era. *Curr Opin Struct Biol* **10:** (3) 371.

Jurecic R, Belmont JW. 2000. University Miami, Sch Med, Dept Microbiol & Immunol, 1600 NW 10th Ave, Miami, Fl 33136, USA. Long distance DD-PCR and cDNA microarrays. *Curr Opin Microbiol* **3:** (3) 316.

Keller B, Feuillet C. 2000. University Zurich, Inst Plant Biol, Dept Plant Mol Biol, Zollikerstr 107, CH-8008 Zurich, Switzerland. Colinearity and gene density in grass genomes (Review). *Trends Plant Sci* **5:** (6) 246.

Kim SH. 2000. UnivCalif, Calvin Lab, Berkeley, Ca 94720, USA. Structural genomics of microbes: An objective. *Curr Opin Struct Biol* **10:** (3) 380.

Koonin EV, Aravind L, Kondrashov AS. 2000. NIH/Natl Lib Med, Natl Ctr Biotechnol Informat, Bethesda, Md 20894, USA. The impact of comparative genomics on our understanding of evolution (Minireview). *Cell* **101:** (6) 573.

Kotra LP, Vakulenko S, Mobashery S*. 2000. *Wayne State Univ, Dept Chem, Detroit, Mi 48202, USA. From genes to sequences to antibiotics: Prospects for future developments from microbial genomics. *Microb Infect* **2:** (6) 651.

Lekven AC, Helde KA, Thorpe CJ, Rooke R, Moon RT. 2000. Univ Washington, Howard Hughes Med Inst, Sch Med, Dept Pharmacol, Ctr Dev Biol, Seattle, Wa 98195, USA. Reverse genetics in zebrafish (Review). *Physiol Genomics* **2:** (2) 37.

Lemaitre B. 2000. CNRS, Ctr Genet Mol, Batiment 26, FR-91198 Gif-sur-Yvette, France. The genome sequence of *Drosophila melanogaster* (Mini-Review) (French, English Abstract). *M S-Med Sci* **16:** (5) 693.

Lewis S, Ashburner M, Reese MG. 2000. Univ Calif, Dept Mol & Cell Biol, Drosophila Genome Project, Berkeley, Ca 94720, USA. Annotating eukaryote genomes. *Curr Opin Struct Biol* **10:** (3) 349.

Lockhart DJ, Winzeler EA. 2000. Novartis Res Fdn, Genome Inst, 3115 Merryfield Row, San Diego, Ca 92121, USA. Genomics, gene expression and DNA arrays (Review). *Nature* **405:** (6788) 827.

Marcotte EM. 2000. UCLA, US/DOE, Inst Mol Biol, Lab Struct Biol & Mol Med, POB 951570, Los Angeles, Ca 90095, USA. Computational genetics: Finding protein function by nonhomology methods. *Curr Opin Struct Biol* **10:** (3) 359.

Marti-Renom MA, Stuart AC, Fiser A, Sanchez R, Melo F, Sali A. 2000. Rockefeller Univ, Pels Family Ctr Biochem & Struct Biol, Labs Mol Biophys, 1230 York Ave, New York, NY 10021, USA. Comparative protein structure modeling of genes and genomes. *Annu Rev Biophys Biomol Struct* **29:** 291.

Moran NA, Wernegreen JJ. 2000. Univ Arizona, Dept Ecol & Evolutionary Biol, Tucson, Az 85721, USA. Lifestyle evolution in symbiotic bacteria: Insights from genomics (Review). *Trends Ecol Evolut* **15:** (8) 321.

Mori H, Isono K, Horiuchi T, Miki T. 2000. Nara Inst Sci & Technol, Res & Educ Ctr Genet Informat, 8916-5 Takayama cho, Ikoma 630 0101, Japan. Functional genomics of *Escherichia coli* in Japan. *Res Microbiol* **151:** (2) 121.

Moult J, Melamud E. 2000. Univ Maryland, Inst Biotechnol, Ctr Adv Res Biotechnol, 8600 Gudelsky Dr, Rockville, Md 20850, USA. From fold to function. *Curr Opin Struct Biol* **10:** (3) 384.

Nierman W, Eisen JA, Fraser CM*. 2000. *Inst Genomic Res, 9712 Med Ctr Dr, Rockville, Md 20850, USA. Microbial genome sequencing 2000: New insights into physiology, evolution and expression analysis. *Res Microbiol* **151:** (2) 79.

Nierman WC, Eisen JA, Fleischmann RD, Fraser CM. 2000. Inst Genomic Res, 9712 Med Ctr Dr, Rockville, Md 20850, USA. Genome data: What do we learn? *Curr Opin Struct Biol* **10:** (3) 343.

Ogasawara N. 2000. Nara Inst Sci & Technol, Grad Sch Biol Sci, 8916-5 Takayama, Nara 630 0101, Japan. Systematic function analysis of *Bacillus subtilis* genes. *Res Microbiol* **151:** (2) 129.

Pandey A, Mann M. 2000. Whitehead Inst Biomed Res, 9 Cambridge Ctr, Cambridge, Ma 02142, USA. Proteomics to study genes and genomes (Review). *Nature* **405:** (6788) 837.

Radman M, Taddei F, Matic I. 2000. Univ Paris 05, INSERM E9916, Fac Med Necker Enfants Malad, 156 rue Vaugirard, FR-75730 Paris 15, France. Evolution-driving genes. *Res Microbiol* **151:** (2) 91.

Risch NJ. 2000. Stanford Univ, Sch Med, Dept Genet, Stanford, Ca 94305, USA. Searching for genetic determinants in the new millennium (Review). *Nature* **405:** (6788) 847.

Rocha EPC, Sekowska A, Danchin A*. 2000. *Univ Hong Kong, Pasteur Res Ctr, Dexter HC Man Bldg, 8 Sassoon Rd, Pokfulam, Hong Kong, Peoples Rep China. Sulphur islands in the *Escherichia coli* genome: Markers of the cell's architecture? (Minireview). *FEBS Lett* **476:** (1-2) 8.

Roses AD. 2000. Glaxo Wellcome PLC, Genet Directorate, Greenford UB6 0HE, England. Pharmacogenetics and the practice of medicine (Review). *Nature* **405:** (6788) 857.

Roth CM, Yarmush ML. 1999. Harvard Univ, MGH, Sch Med, Ctr Engn Med & Surg Serv, Boston, Ma 02114, USA. Nucleic acid biotechnology. *Annu Rev Biomed Eng* **1:** 265.

Skolnick J, Fetrow JS. 2000. Danforth Plant Sci Ctr, Laboratory Computat Genom, 4041 Forest Pk Ave, St Louis, Mo 63108, USA. From genes to protein structure and function: Novel applications of computational approaches in the genomic era (Review). *Trends Biotechnol* **18:** (1) 34.

To KY. 2000. Acad Sinica, Inst Bioagr Sci, Taipei 11529, Taiwan. Identification of differential gene expression by high throughput analysis (Review). *Comb Chem High Throughput Scr* **3:** (3) 235.

Venkatesh B, Gilligan P, Brenner S. 2000. Natl Univ Singapore, Inst Mol & Cell Biol, 30 Med Dr, SG-117609 Singapore, Singapore. Fugu: A compact vertebrate reference genome (Minireview). *FEBS Lett* **476:** (1-2) 3.

Vukmirovic OG, Tilghman SM. 2000. Princeton Univ, Howard Hughes Med Inst, Princeton, NJ 08544, USA. Exploring genome space. *Nature* **405:** (6788) 820.

Walter G, Bussow K, Cahill D, Lueking A, Lehrach H. 2000. Biorchard AS, Nedre Skogvej 14, NO-0281 Oslo, Norway. Protein arrays for gene expression and molecular interaction screening. *Curr Opin Microbiol* **3:** (3) 298.

Waring JF, Ulrich RG. 2000. Abbott Labs, Strateg & Exploratory Sci, Abbott Pk, Il 60064, USA. The impact of genomics-based technologies on drug safety evaluation. *Annu Rev Pharmacol Toxicol* **40:** 335.

Washburn MP, Yates JR. 2000. Univ Washington, Dept Mol Biotechnol, Seattle, Wa 98195, USA. Analysis of the microbial proteome. *Curr Opin Microbiol* **3:** (3) 292.

Weinstock GM, Smajs D, Hardham J, Norris SJ. 2000. Univ Texas, Med Sch, Dept Microbiol & Mol Genet, 6431 Fannin St, Houston, Tx 77030, USA. From microbial genome sequence to applications. *Res Microbiol* **151:** (2) 151.

Wittliff JL, Kunitake ST, Chu SS, Travis JC. 2000. Univ Louisville, James Graham Brown Canc Ctr, Hormone Receptor Lab, Room 421, 529 Sth Jackson St, Louisville, Ky 40202, USA. Applications of laser capture microdissection in genomics and proteomics (Review). *J Clin Ligand Assay* **23:** (1) 66.

Copyright © 2001 John Wiley & Sons, Ltd. 

### 2 General

Lukowitz W, Gillmor CS, Scheible WR. 2000. Carnegie Inst Washington, Dept Plant Biol, 260 Panama St, Stanford, Ca 94305, USA. Positional cloning in *Arabidopsis*. Why it feels good to have a genome initiative working for you. *Plant Physiol* **123:** (3) 795.

### 3 Large-scale sequencing and mapping

Brugere JF, Cornillot E*, Metenier G, Bensimon A, Vivares CP. 2000. *Univ Blaise Pascal, UPRES A CNRS 6023, Equipe Parasitol Mol & Cellulaire, FR-63177 Aubiere, France. *Encephalitozoon cuniculi* (microspora) genome: Physical map and evidence for telomere-associated rDNA units on all chromosomes. *Nucleic Acids Res* **28:** (10) 2026.

Chesnick JM, Goff M, Graham J, Ocampo C, Lang BF, Seif E, Burger G*. 2000. *Univ Montreal, Dept Biochim, 2900 Blvd Edouard Montpetit, Montreal, Quebec, Canada H3T 1J4. The mitochondrial genome of the stramenopile alga *Chrysodidymus synuroideus*. Complete sequence, gene content and genome organization. *Nucleic Acids Res* **28:** (13) 2512.

Haring D, Kypr J*. 2000. *Acad Sci Czech Republ, Inst Biophys, Kralovopolska 135, CZ-61265 Brno, Czech Republic. *Escherichia coli* genome is composed of twin distinct types of nucleotide sequences. *Biochem Biophys Res Commun* **272:** (2) 571.

Horvath JE, Schwartz S, Eichler EE*. 2000. *Case Western Reserve Univ, Sch Med, Dept Genet, Cleveland, Oh 44106, USA. The mosaic structure of human pericentromeric DNA: A strategy for characterizing complex regions of the human genome. *Genome Res* **10:** (6) 839.

Klein PE, Klien RR, Cartinhour SW, Ulanch PE, Dong JM, Obert JA, Morishige DT, Schlueter SD, Childs KL, Ale M, Mullet JE*. 2000. *Texas A&M Univ, Crop Biotechnol Ctr, College Station, Tx 77843, USA. A high-throughput AFLP-based method for constructing integrated genetic and physical maps: Progress toward a sorghum genome map. *Genome Res* **10:** (6) 789.

Lee LF, Wu P, Sui DX, Ren DL, Kamil J, Kung HJ, Witter RL. 2000. USDA/ARS, Avian Dis & Oncol Lab, 3606 East Mt Hope Rd, East Lansing, Mi 48823, USA. The complete unique long sequence and the overall genomic organization of the GA strain of Marek's disease virus. *Proc Natl Acad Sci U S A* **97:** (11) 6091.

Liang F, Holt I, Pertea G, Karamycheva S, Salzberg SL, Quackenbush J*. 2000. *Inst Genomic Res, Rockville, Md, USA. Gene Index analysis of the human genome estimates approximately 120,000 genes. *Nat Genet* **25:** (2) 239.

Lockhart BE, Menke J, Dahal G, Olszewski NE. 2000. Univ Minnesota, Dept Plant Pathol, St Paul, Mn 55108, USA. Characterization and genomic analysis of tobacco vein clearing virus, a plant pararetrovirus that is transmitted vertically and related to sequences integrated in the host genome. *J Gen Virol* **810:** (6) 1579.

Mao L, Wood TC, Yu YS, Budiman MA, Tomkins J, Woo SS, Sasinowski M, Presting G, Frisch D, Goff S, Dean RA, Wing RA*. 2000. *Clemson Univ, Genom Inst, Clemson, SC 29634, USA. Rice transposable elements: A survey of 73,000 sequence-tagged-connectors. *Genome Res* **10:** (7) 982.

Nedelcu AM, Lee RW, Lemieux C, Gray MW, Burger G. 2000. Univ Arizona, Dept Ecol & Evolutionary Biol, Tucson, Az 85721, USA. The complete mitochondrial DNA sequence of *Scenedesmus obliquus* reflects an intermediate stage in the evolution of the green algal mitochondrial genome. *Genome Res* **10:** (6) 819.

Palfrey D, Picardo M, Hine AV*. 2000. *Aston Univ, Pharmaceut Sci Res Inst, Aston Triangle, Birmingham B4 7ET, England. A new randomization assay reveals unexpected elements of sequence bias in model 'randomized' gene libraries: Implications for biopanning. *Gene* **251:** (1) 91.

Roach JC, Thorsson V, Siegel AF. 2000. Inst Syst Biol, 4225 Roosevelt Way NE, Suite 200, Seattle, Wa 98105, USA. Parking strategies for genome sequencing. *Genome Res* **10:** (7) 1020.

Simpson AJG, Reinach FC, Arruda P *et al*. 2000. c/o Setubal JC, Univ Estadual Campinas, Inst Computacao, CP 6176, BR-13083-970 Campinas, SP, Brazil. The genome sequence of the plant pathogen *Xylella fastidiosa*. *Nature* **406:** (6792) 151.

Wernegreen JJ, Ochman H, Jones IB, Moran NA. 2000. Marine Biol Lab, Ctr Comparat Mol Biol & Evolut, 7 MBL St, Woods Hole, Ma 02543, USA. Decoupling of genome size and sequence divergence in a symbiotic bacterium. *J Bacteriol* **182:** (13) 3867.

### 4 Genome evolution

Arnason U, Gullberg A, Gretarsdottir S, Ursing B, Janke A. 2000. Lund Univ, Dept Genet, Div Evolutionary Mol Systemat, Solvegatan 29, SE-22362 Lund, Sweden. The mitochondrial genome of the sperm whale and a new molecular reference for estimating eutheria divergence dates. *J Mol Evol* **50:** (6) 569.

Fedoroff N. 2000. Penn State Univ, University Park, Pa 16803, USA. Transposons and genome evolution in plants. *Proc Natl Acad Sci U S A* **97:** (13) 7002.

Gaut BS, D'Ennequin ML, Peek AS, Sawkins MC. 2000. Univ Calif, Dept Ecol & Evolutionary Biol, Irvine, Ca 92697, USA. Maize as a model for the evolution of plant nuclear genomes. *Proc Natl Acad Sci U S A* **97:** (13) 7008.

Gierlik A, Kowalczuk M, Mackiewicz P, Dudek MR, Cebrat S*. 2000. *Univ Wroclaw, Inst Microbiol, ul Przybyszewskiego 63-77, PL-54148 Wroclaw, Poland. Is there replication-associated mutational pressure in the *Saccharomyces cerevisiae* genome? *J Theor Biol* **202:** (4) 305.

Hughes TR, Roberts CJ, Dai HY, Jones AR, Meyer MR, Slade D, Burchard J, Dow S, Ward TR, Kidd MJ, Marton MJ*. 2000. *Rosetta Inpharmatics Inc, Kirkland, Wa, USA. Widespread aneuploidy revealed by DNA microarray expression profiling. *Nat Genet* **25:** (3) 333.

Kalendar R, Tanskanen J, Immonen S, Nevo E, Schulman AH*. 2000. *Univ Helsinki, Inst Biotechnol, Plant Genomics Lab, Viikki Bioctr, POB 56, FI-00014 Helsinki, Finland. Genome evolution of wild barley (*Hordeum spontaneum*) by *BARE*-1 retrotransposon dynamics in response to sharp microclimatic divergence. *Proc Natl Acad Sci U S A* **97:** (12) 6603.

Kulski JK, Gaudieri S, Dawkins RL. 2000. Univ Western Australia, Fac Med & Dent, Ctr Mol Immunol & Instrumentat, POB 5100, Canning Vale, WA 6155, Australia. Using *Alu-J* elements as molecular clocks to trace the evolutionary relationships between duplicated HLA class I genomic segments. *J Mol Evol* **50:** (6) 510.

Le QH, Wright S, Yu ZH, Bureau T*. 2000. *McGill Univ, Dept Biol, 1205 Doctor Penfield Ave, Montreal, Quebec, Canada H3A 1B1. Transposon diversity in *Arabidopsis thaliana*. *Proc Natl Acad Sci U S A* **97:** (13) 7376.

Marin I, Llorens C. 2000. Univ Valencia, Dept Genet, Calle Doctor Moliner 50, ES-46100 Valencia, Spain. Ty3/*gypsy* retrotransposons: Description of new *Arabidopsis thaliana* elements and evolutionary perspectives derived from comparative genomic data. *Mol Biol Evol* **17:** (7) 1040.

Palmer JD, Adams KL, Cho YR, Parkinson CL, Qiu YL, Song KM. 2000. Indiana Univ, Dept Biol, 1001 East 3rd St, Bloomington, In 47405, USA. Dynamic evolution of plant mitochondrial genomes: Mobile genes and introns and highly variable mutation rates. *Proc Natl Acad Sci U S A* **97:** (13) 6960.

Pizzi E, Frontali C*. 2000. *Ist Super Sanita, Cell Biol Lab, Viale Regina Elena 299, IT-00161 Rome, Italy. Divergence of noncoding sequences and of insertions encoding nonglobular domains at a genomic region well conserved in plasmodia. *J Mol Evol* **50:** (5) 474.

Wilson K, Cahill V, Ballment E, Benzie J. 2000. Australian Inst Marine Sci, PMB 3, Townsville, Qld 4810, Australia. The complete sequence

Copyright © 2001 John Wiley & Sons, Ltd.

of the mitochondrial genome of the crustacean *Penaeus monodon*: Are malacostracan crustaceans more closely related to insects than to branchiopods? *Mol Biol Evol* **17:** (6) 863.

Wolf YI, Kondrashov FA, Koonin EV. 2000. NIH, Natl Ctr Biotechnol Informat, Bethesda, Md 20894, USA. No footprints of primordial introns in a eukaryotic genome. *Trends Genet* **16:** (8) 333.

Yamaguchi J, Devare SG, Brennan CA*. 2000. *Abbott Labs, AIDS Res & Retrovirus Discovery, 100 Abbott Pk Rd, Abbott Park, Il 60064, USA. Identification of a new HIV-2 subtype based on phylogenetic analysis of full-length genomic sequence. *AIDS Res Hum Retroviruses* **16:** (9) 925.

### 5 Comparative genomics

Alm RA, Bina J, Andrews BM, Doig P, Hancock REW, Trust TJ. 2000. Infect Discovery AstraZeneca R&D Boston, 35 Gatehouse Dr, Waltham, Ma 02451, USA. Comparative genomics of *Helicobacter pylori*: Analysis of the outer membrane protein families. *Infect Immun* **68:** (7) 4155.

Ballard JWO. 2000. Field Museum Nat Hist, 1400 Sth Lake Shore Dr, Chicago, Il 60605, USA. Comparative genomics of mitochondrial DNA in members of the *Drosophila melanogaster* subgroup. *J Mol Evol* **51:** (1) 48.

Ballard JWO. 2000. Address as above. Comparative genomics of mitochondrial DNA in *Drosophila simulans*. *J Mol Evol* **51:** (1) 64.

Batzoglou S, Pachter L, Mesirov JP, Berger B, Lander ES*. 2000. *Univ Calif, Dept Math, Berkeley, Ca 94720, USA. Human and mouse gene structure: Comparative analysis and application to exon prediction. *Genome Res* **10:** (7) 950.

Bradel BG, Preil W, Jeske H*. 2000. *Univ Stuttgart, Inst Biol, Abt Mol Biol & Virol Pflanzen, Pfaffenwaldring 57, DE-70550 Stuttgart, Germany. Sequence analysis and genome organisation of *poinsettia* mosaic virus (PnMV) reveal closer relationship to marafiviruses than to tymoviruses. *Virology* **271:** (2) 289.

Crollius HR, Jaillon O, Bernot A, Dasilva C, Bouneau L, Fischer C, Fizames C, Wincker P, Brottier P, Quetier F, Saurin W, Weissenbach J*. 2000. *Genoscope, Evry, France. Estimate of human gene number provided by genome-wide analysis using *Tetraodon nigroviridis* DNA sequence. *Nat Genet* **25:** (2) 235.

Devos KM, Gale MD. 2000. John Innes Ctr Plant Sci Res, Norwich Res Pk, Norwich NR4 7UH, England. Genome relationships: The grass model in current research. *Plant Cell* **12:** (5) 637.

Fortini ME, Skupski MP, Boguski MS, Hariharan IK. 2000. Univ Penn, Sch Med, Dept Genet, Stellar Chance Labs, 422 Curie Blvd, Philadelphia, Pa 19104, USA. A survey of human disease gene counterparts in the *Drosophila* genome. *J Cell Biol* **150:** (2) F23.

Heslop-Harrison JS. 2000. John Innes Ctr Plant Sci Res, Norwich NR4 7UH, England. Comparative genome organization in plants: From sequence and markers to chromatin and chromosomes. *Plant Cell* **12:** (5) 617.

Lan TH, Del Monte TA, Reischmann KP, Hyman J, Kowalski SP, McFerson J, Kresovich S, Paterson AH*. 2000. *Texas A&M Univ, Dept Soil & Crop Sci, College Station, Tx 77843, USA. An EST-enriched comparative map of *Brassica oleracea* and *Arabidopsis thaliana*. *Genome Res* **10:** (6) 776.

Lin J, Gerstein M*. 2000. *Yale Univ, Dept Mol Biophys & Biochem, POB 6666, New Haven, Ct 06520, USA. Whole-genome trees based on the occurrence of folds and orthologs: Implications for comparing genomes on different levels. *Genome Res* **10:** (6) 808.

Mallon AM, Platzer M, Bate R, Gloeckner G, Botcherby MRM, Nordsiek G, Strivens MA, Kioschis P, Dangel A, Cunningham D, Straw RVA, Weston P, Gilbert M, Fernando S, Goodall K, Hunter G, Greystrong JS, Clarke D, Kimberley C, Goerdes M, Blechschmidt K, Rump A, Hinzmann B, Mundy CR, Miller W, Poustka A, Herman GE, Rhodes M, Denny P, Rosenthal A, Brown SDM. 2000. MRC UK Mouse Genome Ctr, Harwell, Oxon, England. Comparative genome sequence analysis of the Bpa/Str region in mouse and man. *Genome Res* **10:** (6) 758.

Shirai M, Hirakawa H, Kimoto M, Tabuchi M, Kishi F, Ouchi K, Shiba T, Ishii K, Hattori M, Kuhara S, Nakazawa T. 2000. Yamaguchi Univ, Sch Med, Dept Microbiol, Minamikogushi 1-1-1, Yamaguchi 755 8505, Japan. Comparison of whole genome sequences of *Chlamydia pneumoniae* J138 from Japan and CWL029 from USA. *Nucleic Acids Res* **28:** (12) 2311.

### 6 Pathways, gene families and regulons

Dillon N, Sabbattini P. 2000. Hammersmith Hosp, Imperial Coll, Sch Med, Dept Biotechnol, Ctr Clin Sci, MRC Gene Regulation & Chromatin Grp, Ducane Rd, London W12 0NN, England. Functional gene expression domains: Defining the functional unit of eukaryotic gene regulation. *Bioessays* **22:** (7) 657.

Littleton JT. 2000. MIT, Ctr Learning & Memory, Dept Biol, 50 Ames St, Cambridge, Ma 02139, USA. A genomic analysis of membrane trafficking and neurotransmitter release in *Drosophila*. *J Cell Biol* **150:** (2) F77.

Lyons TJ, Gasch AP, Gaither LA, Botstein D, Brown PO, Eide DJ*. 2000. *Univ Missouri, Dept Nutr Sci, Columbia, Mo 65211, USA. Genome-wide characterization of the Zap1p zinc-responsive regulon in yeast. *Proc Natl Acad Sci U S A* **97:** (14) 7957.

Murai K, Miyake K*, Andoh J, Iijima S. 2000. *Nagoya Univ, Grad Sch Engn, Dept Biotechnol, Chikusa ku, Furo cho, Nagoya, Aichi 464 8603, Japan. Transcriptional regulation of *nir* and *nor* operons of *Paracoccus denitrificans*. *J Biosci Bioeng* **89:** (4) 384.

Paquette SM, Bak S, Feyereisen R*. 2000. *Univ Arizona, Dept Entomol, 1140 East South Campus Dr, Tucson, Az 85721, USA. Intron-exon organization and phylogeny in a large superfamily, the paralogous cytochrome P450 genes of *Arabidopsis thaliana*. *DNA Cell Biol* **19:** (5) 307.

Salgado H, Moreno-Hagelsieb G, Smith TF, Collado-Vides J*. 2000. *Univ Nacl Autonoma Mexico, Ctr Invest Fijacion Nitrogeno, AP 565-A, MX-62100 Cuernavaca, Morelos, Mexico. Operons in *Escherichia coli*: Genomic analyses and predictions. *Proc Natl Acad Sci U S A* **97:** (12) 6652.

Stevens TJ, Arkin IT*. 2000. *Univ Cambridge, Ctr Mol Recognit, Dept Biol, 80 Tennis Court Rd, Cambridge CB2 1GA, England. Do more complex organisms have a greater proportion of membrane proteins in their genomes? *Protein-Struct Funct Genet* **39:** (4) 417.

### 7 Pharmacogenomics

Fan JB, Chen XQ, Halushka MK, Berno A, Huang XH, Ryder T, Lipshutz RJ*, Lockhart DJ, Chakravarti A. 2000. *Illumina Inc, San Diego, Ca 92121, USA. Parallel genotyping of human SNPs using generic high-density oligonucleotide tag arrays. *Genome Res* **10:** (6) 853.

Pastinen T, Raitio M, Lindroos K, Tainola P, Peltonen L, Syvanen AC. 2000. McGill Univ, Ctr Hlth, Montreal Genome Ctr, Room C10-133, 1650 Cedar Ave, Montreal, Quebec, Canada H3G 1A4. A system for specific, high-throughput genotyping by allele-specific primer extension on microarrays. *Genome Res* **10:** (7) 1031.

Woolley AT, Guillemette C, Cheung CL, Housman DE, Lieber CM*. 2000. *MIT, Ctr Canc Res, Cambridge, Ma 02139, USA. Direct haplotyping of kilobase-size DNA using carbon nanotube probes. *Nat Biotechnol* **18:** (7) 760.

### 8 Large-scale mutagenesis programmes

Dickinson P, Kimber WL, Kilanowski FM, Webb S, Stevenson BJ,

Copyright © 2001 John Wiley & Sons, Ltd. 

Porteous DJ, Dorin JR*. 2000. *Western Gen Hosp, MRC Human Genet Unit, Crewe Rd, Edinburgh EH4 2XU, Scotland. Enhancing the efficiency of introducing precise mutations into the mouse genome by hit and run gene targeting. *Transgenic Res* **9:** (1) 55.

Jeon JS, Lee S, Jung KH, Jun SH, Jeong DH, Lee J, Kim C, Jang S, Lee S, Yang K, Nam J, An K, Han MJ, Sung RJ, Choi HS, Yu JH, Choi JH, Cho SY, Cha SS, Kim SI, An G*. 2000. *Univ Sci & Technol, Natl Res Lab Plant Funct Genomics, Div Mol & Life Sci, Pohang 790784, South Korea. T-DNA insertional mutagenesis for functional genomics in rice. *Plant J* **22:** (6) 561.

McCallum CM, Comai L, Greene EA, Henikoff S*. 2000. *Fred Hutchinson Canc Res Ctr, Div Basic Sci, Seattle, Wa 98109, USA. Targeting Induced Local Lesions In Genomes (TILLING) for plant functional genomics. *Plant Physiol* **123:** (2) 439.

Toh-e A, Oguchi T. 2000. Univ Tokyo, Grad Sch Sci, Dept Biol Sci, Hongo, Tokyo 113 0033, Japan. An improved integration replacement/disruption method for mutagenesis of yeast essential genes. *Genes Genet Syst* **75:** (1) 33.

### 9 Functional genomics

Alimi JP, Poirot O, Lopez F, Claverie JM*. 2000. CNRS/UMR 1889, Lab Struct & Genet Informat, FR-13402 Marseille 20, France. Reverse transcriptase-polymerase chain reaction validation of 25 “orphan” genes from *Escherichia coli* K-12 MG1655. *Genome Res* **10:** (7) 959.

Backen AC, Broadbent ID, Fetherston RW, Rosamond JDC, Schnell NF, Stark MJR*. 2000. *Univ Dundee, Dept Biochem, MSI/WTB Complex, Dundee DD1 5EH, Scotland. Evaluation of the *CaMAL2* promoter for regulated expression of genes in *Candida albicans*. *Yeast* **16:** (12) 1121.

Fearns R, Collins PL*, Peeples ME. 2000. *NIH/NIAID, Bldg 7, Rm 100, 7 Ctr Dr, MSC 0720, Bethesda, Md 20892, USA. Functional analysis of the genomic and antigenomic promoters of human respiratory syncytial virus. *J Virol* **74:** (13) 6006.

Horecka J, Jigami Y. 2000. AIST, NIBH, Dept Molec Biol, 1-1 Higashi, Tsukuba, Ibaraki 305-8566, Japan. Identifying tagged transposon insertion sites in yeast by direct genomic sequencing. *Yeast* **16:** (10) 967.

Kihara D, Kanehisa M*. 2000. *Kyoto Univ, Inst Chem Res, Uji, Kyoto 611 0011, Japan. Tandem clusters of membrane proteins in complete genome sequences. *Genome Res* **10:** (6) 731.

Larsson M, Graslund S, Li YB, Brundell E, Uhlen M, Hoog C, Stahl S*. 2000. *Royal Inst Technol, KTH, Dept Biotechnol, SE-10044 Stockholm, Sweden. High-throughput protein expression of cDNA products as a tool in functional genomics. *J Biotechnol* **80:** (2) 143.

Moreno MB, Duran A, Ribas JC*. 2000. *Univ Salamanca/CSIC, Inst Microbiol Bioquim, Dept Microbiol & Genet, Campus Miguel Unamuno, Edifico Dept, Rm 222, ES-37007 Salamanca, Spain. A family of multifunctional thiamine-repressible expression vectors for fission yeast. *Yeast* **16:** (9) 861.

Murray BW, Sultmann H, Klein J*. 2000. *Max-Planck-Inst Biol, Immunogenet Abt, Corrensstr 42, DE-72076 Tubingen, Germany. Identification and linkage of the proteasome activator complex PA28 subunit genes in zebrafish. *Scand J Immunol* **51:** (6) 571.

Pelling AL, Thorne AW, Crane-Robinson C*. 2000. *Univ Portsmouth, Fac Sci, Inst Biomed & Biomol Sci, Biophys Lab, Portsmouth PO1 2DT, England. A human genomic library enriched in transcriptionally active sequences (a DNA library). *Genome Res* **10:** (6) 874.

Pinson B, Sagot I, Daignan-Fornier B*. 2000. *CNRS UPR 9026, Inst Biochim & Genet Cellulaires, 1 rue Camille St Saens, FR-33077 Bordeaux, France. Identification of genes affecting selenite toxicity and resistance in *Saccharomyces cerevisiae*. *Mol Microbiol* **36:** (3) 679.

Underhill DA, Vogan KJ, Kibar Z, Morrison J, Rommens J, Gros P. 2000. Univ Alberta, Dept Med Genet, 8-29 Med Sci Bldg, Edmonton, Alberta, Canada T6G 2H7. Transcription mapping and expression analysis of candidate genes in the vicinity of the mouse *loop-tail* mutation. *Mamm Genome* **11:** (8) 633.

Unsworth KE, Holden DW*. 2000. *Hammersmith Hosp, Imperial Coll, Sch Med, Dept Infect Dis, Du Cane Rd, London W12 0NN, England. Identification and analysis of bacterial virulence genes *in vivo*. *Philos Trans R Soc Lond B* **355:** (1397) 613.

Warrington JA, Nair A, Mahadevappa M, Tsyganskaya M. 2000. Affymetrix Inc, 3380 Cent Expressway, Santa Clara, Ca 95051, USA. Comparison of human adult and fetal expression and identification of 535 housekeeping/maintenance genes. *Physiol Genomics* **2:** (3) 143.

### 10 Transcriptomics

Ewing B, Green P*. 2000. *Univ Washington, Dept Mol Biotechnol, Seattle, Wa 98195, USA. Analysis of expressed sequence tags indicates 35,000 human genes. *Nat Genet* **25:** (2) 232.

Hwang DM, Dempsey AA, Lee CY*, Liew CC. 2000. *Univ Toronto, Banting Inst, Dept Lab Med & Pathobiol, 100 Coll St, Toronto, Ontario, Canada M5G 1L5. Identification of differentially expressed genes in cardiac hypertrophy by analysis of expressed sequence tags. *Genomics* **66:** (1) 1.

Hyodo H, Takemura M, Yokota A, Ohyama K, Kohchi T*. 2000. *Nara Inst Sci & Technol, Grad Sch Biol Sci, 8916-5 Takayama, Nara 630 0101, Japan. Systematic isolation of highly transcribed genes in inflorescence apices in *Arabidopsis thaliana* from an equalized cDNA library. *Biosci Biotechnol Biochem* **64:** (7) 1538.

Ingram GC, Boisnard-Lorig C, Dumas C, Rogowsky PM*. 2000. *Univ Lyon 1, ENSL, INRA-CNRS, UMR5667, RDP, ENS-Lyon, 46 Allee Italie, FR-69364 Lyon 07, France. Expression patterns of genes encoding HD-ZipIV homeo domain proteins define specific domains in maize embryos and meristems. *Plant J* **22:** (5) 401.

Kaushik N, Malaspina A, De Belleroche J*. 2000. *Univ London, Imperial Coll Sci Technol & Med, Dept Neuromuscular Dis, Div Neurosci & Psychol Med, London W6 8RF, England. Characterization of trinucleotide- and tandem repeat-containing transcripts obtained from human spinal cord cDNA library by high-density filter hybridization. *DNA Cell Biol* **19:** (5) 265.

Lee CK, Weindruch R*, Prolla TA. 2000. *Univ Wisconsin, Dept Med, Madison, Wi 53706, USA. Gene-expression profile of the ageing brain in mice. *Nat Genet* **25:** (3) 294.

Lyn D, Liu XW, Bennett NA, Emmett NL. 2000. Morehouse Sch Med, Dept Biochem, 720 Westview Dr, Atlanta, Ga 30310, USA. Gene expression profile in mouse myocardium after ischemia. *Physiol Genomics* **2:** (3) 93.

Merrell DS, Camilli A*. 2000. *Tufts Univ, Sch Med, Dept Mol Biol & Microbiol, 136 Harrison Ave, Boston, Ma 02111, USA. Detection and analysis of gene expression during infection by *in vivo* expression technology. *Philos Trans R Soc Lond B* **355:** (1397) 587.

Michiels EMC, Oussoren E, Van Groenigen M, Pauws E, Bossuyt PMM, Voute PA, Baas F*. 1999. *Acad Med Ctr, Neurozintuigen Lab, POB 22700, NL-1100 DE Amsterdam, The Netherlands. Genes differentially expressed in medulloblastoma and fetal brain. *Physiol Genomics* **1:** (2) 83.

Nelson PS, Han D, Rochon Y, Corthals GL, Lin BY, Monson A, Nguyen V, Franza BR, Plymate SR, Aebersold R, Hood L. 2000. Fred Hutchinson Canc Res Ctr, Div Human Biol, Mailstop D4-100, 1100 Fairview Ave, Seattle, Wa 98109, USA. Comprehensive analyses of prostate gene expression: Convergence of expressed sequence tag databases, transcript profiling and proteomics. *Electrophoresis* **21:** (9) 1823.

Page N, Butlin D, Manyonda I, Lowry P. 2000. Univ Reading, Sch Anim & Microbial Sci, Reading RG6 6AJ, England. The development of a genetic profile of placental gene expression during the first trimester of pregnancy: A potential tool for identifying novel secreted markers. *Fetal Diagn Ther* **15:** (4) 237.

Posas F, Chambers JR, Heyman JA, Hoeffler JP, De Nadal E, Arino J*.

Copyright © 2001 John Wiley & Sons, Ltd. 

2000. *Univ Autonoma Barcelona, Fac Vet, Dept Bioquim & Biol Mol, ES-08193 Barcelona, Spain. The transcriptional response of yeast to saline stress. *J Biol Chem* **275:** (23) 17249.

Sato H, Sagai M, Suzuki KT, Aoki Y*. 1999. *Natl Inst Environm Studies, Environm Hlth Sci Div, Ibaraki, Osaka 305 0053, Japan. Identification, by cDNA microarray, of A-raf and proliferating cell nuclear antigen as genes induced in rat lung by exposure to diesel exhaust. *Res Commun Mol Pathol Pharmacol* **105:** (1-2) 77.

Smid-Koopman E, Blok LJ, Chadha-Aiwani S, Helmerhorst TJM, Brinkmann AO, Huikeshoven FJ. 2000. Univ Rotterdam Hosp, Dept Obstet & Gynaecol, POB 2040, NL-3000 CA Rotterdam, The Netherlands. Gene expression profiles of human endometrial cancer samples using a cDNA-expression array technique: Assessment of an analysis method. *Br J Cancer* **83:** (2) 246.

Soriano MA, Tessier M, Certa U, Gill R*. 2000. *F Hoffmann La Roche & Co Ltd, Preclin CNS Res, PRPN, Bldg 68-410, CH-4070 Basel, Switzerland. Parallel gene expression monitoring using oligonucleotide probe arrays of multiple transcripts with an animal model of focal ischemia. *J Cereb Blood Flow Metab* **20:** (7) 1045.

Stanton LW, Garrard LJ, Damm D, Garrick BL, Lam A, Kapoun AM, Zheng Q, Protter AA, Schreiner GF, White RT*. 2000. *Scios Inc, 820 West Maude Ave, Sunnyvale, Ca 94086, USA. Altered patterns of gene expression in response to myocardial infarction. *Circ Res* **86:** (9) 939.

Sun M, Yang C, Yin L, Shupe T, Ilic Z, Fridreich T, Sell D. 2000. Albany Med Coll, Dept Pathol & Lab Med, Albany, NY 12208, USA. Preliminary microarray analysis of differences in mRNAs of cell cycle control and carcinogen metabolizing enzymes in normal and p53 null mice. *J Tumor Marker Oncol* **15:** (1) 77.

Van der Biezen EA, Juwana H, Parker JE, Jones JDG*. 2000. *John Innes Ctr Plant Sci Res, Sainsbury Lab, Norwich Res Pk, Norwich NR4 7UH, England. cDNA-AFLP display for the isolation of *Peronospora parasitica* genes expressed during infection in *Arabidopsis thaliana*. *Mol Plant Microbe Interact* **13:** (8) 895.

Wellmann A, Thieblemont C, Pittaluga S, Sakai A, Jaffe ES, Siebert P, Raffeld M*. 2000. *NIH/NCI, Pathol Lab, Hematopathol Sect, Bldg 10, Rm 2N110, 9000 Rockville Pike, Bethesda, Md 20892, USA. Detection of differentially expressed genes in lymphomas using cDNA arrays: Identification of clusterin as a new diagnostic marker for anaplastic large-cell lymphomas. *Blood* **96:** (2) 398.

### 11 Proteomics

Anglade P, Demey E, Labas V, Le Caer JP, Chich JF*. 2000. *INRA URBSP, Domaine Vilbert, Bat 526, FR-78352 Jouy-en-Josas, France. Towards a proteomic map of *Lactococcus lactis* NCDO 763. *Electrophoresis* **21:** (12) 2546.

Araki N, Morimasa T, Sakai T, Tokuoh H, Yunoue S, Kamo M, Miyazaki K, Abe K, Saya H, Tsugita A. 2000. Kumamoto Univ, Sch Med, Dept Tumor Genet & Biol, Kumamoto 860 0811, Japan. Comparative analysis of brain proteins from p53-deficient mice by two-dimensional electrophoresis. *Electrophoresis* **21:** (9) 1880.

Bon E, Recordon-Navarro P, Durrens P*, Iwase M, Toh-e A, Aigle M. 2000. *IBGC, Lab Biol Cell Levure, 1 rue Camille Saint-Saens, FR-33077 Bordeaux, France. A network of proteins around Rvs167p and Rvs161p, two proteins related to the yeast actin cytoskeleton. *Yeast* **16:** (13) 1229.

Chu PW, Yap MN, Wu CY, Huang CM, Pan FM, Tseng MJ, Chen ST*. 2000. *Inst Biol Chem, Acad Sinica, Taipei, Taiwan. A proteomic analysis of secreted proteins from xylan-induced *Bacillus* sp strain K1. *Electrophoresis* **21:** (9) 1740.

Cole AR, Ji H, Simpson RJ*. 2000. *Royal Melbourne Hosp, Ludwig Inst Canc Res, Joint Prot Struct Lab, Melbourne Branch, POB 2008, Melbourne, Vic 3050, Australia. Proteomic analysis of colonic crypts from normal, multiple intestinal neoplasia and p53-null mice: A comparison with colonic polyps. *Electrophoresis* **21:** (9) 1772.

Englert C, Moradpour D, Blum HE. 2000. Forschungszentrum Karlsruhe, Inst Genet & Toxikol, Hermann von Helmholtz Pl 1, DE-76344 Eggenstein, Germany. Proteomics (German). *Dtsch Med Wochenschr* **125:** (25-26) 803.

Iwahashi Y, Hosoda H. 2000. Natl Food Res Inst, Minist Agr Forestry & Fisheries, Kannondai 1 chome, Tsukuba, Ibaraki 305 8642, Japan. Effect of heat stress on tomato fruit protein expression. *Electrophoresis* **21:** (9) 1766.

Joubert-Caron R, Le Caer JP, Montandon F, Poirier F, Pontet M, Imam N, Feuillard J, Bladier D, Rossier J, Caron M. 2000. UFR Leonard de Vinci, Lab Biochim Prot & Proteomie, EA 1625, 74 rue Marcel Cachin, FR-93017 Bobigny, France. Protein analysis by mass spectrometry and sequence database searching: A proteomic approach to identify human lymphoblastoid cell line proteins. *Electrophoresis* **21:** (12) 2566.

Kaji H, Tsuji T, Mawuenyega KG, Wakamiya A, Taoka M, Isobe T. 2000. Tokyo Metropolitan Univ, Grad Sch Sci, Dept Chem, Biochem Lab, Minami Osawa 1-1, Hachioji, Tokyo 192 0397, Japan. Profiling of *Caenorhabditis elegans* proteins using two-dimensional gel electrophoresis and matrix assisted laser desorption/ionization-time-of-flight-mass spectrometry. *Electrophoresis* **21:** (9) 1755.

Kanaya S, Ujiie Y, Hasegawa K, Sato T, Imada H, Kinouchi M, Kudo Y, Ogata T, Ohya H, Kamada H, Itamoto K, Katsura K. 2000. Yamagata Univ, Fac Engn, Dept Elect Informat & Engn, Jounan 4-3-16, Yonezawa, Yamagata 992 851, Japan. Proteome analysis of *Oncorhynchus* species during embryogenesis. *Electrophoresis* **21:** (9) 1907.

Kawakami T, Nagata T, Muraguchi A, Nishimura T*. 2000. *Glaxo Wellcome KK, Core Technol Dept, Proteomics Unit, Tsukuba Res Labs, Div Res, 43 Wadai, Tsukuba, Ibaraki 300 4247, Japan. Alteration of protein composition in mouse thymocytes by signals through T-cell receptor. *Electrophoresis* **21:** (9) 1846.

Kumar VB, Franko MP, Farr SA, Armbrecht HJ, Morley JE. 2000. Vet Adm Med Ctr, Ctr Geriatr Res Educ & Clin, St Louis, Mo 63125, USA. Identification of age-dependent changes in expression of senescence-accelerated mouse (SAMP8) hippocampal proteins by expression array analysis. *Biochem Biophys Res Commun* **272:** (3) 657.

Locke S, Figeys D*. 2000. *MDS Ocata, 600 Univ Ave, Suite 1075, Toronto, Ontario, Canada M5G 1X5. Techniques for the optimization of proteomic strategies based on head column stacking capillary electrophoresis. *Anal Chem* **72:** (13) 2684.

Macri J, McGee B, Thomas JN, Du P, Stevenson TI, Kilby GW, Rapundalo ST*. 2000. *Parke Davis Pharmaceut Res, Dept Biochem, 2800 Plymouth Rd, Ann Arbor, Mi 48105, USA. Cardiac sarcoplasmic reticulum and sarcolemmal proteins separated by two-dimensional electrophoresis: Surfactant effects on membrane solubilization. *Electrophoresis* **21:** (9) 1685.

Minowa T, Ohtsuka S, Sasai H, Kamada M. 2000. Japan Tobacco Inc, Cent Pharmaceut Res Inst, Pharmaceut Frontier Res Labs, 13-2 Fukuura 1 chome, Yokohama, Kanagawa 236 000, Japan. Proteomic analysis of the small intestine and colon epithelia of adenomatous polyposis coli gene-mutant mice by two-dimensional gel electrophoresis. *Electrophoresis* **21:** (9) 1782.

Nakagawa M, Yamagaki T, Nakanishi H*. 2000. *AIST, Natl Inst Biosci & Human Technol, 1-1 Higashi, Tsukuba, Ibaraki 305 8566, Japan. Fluorescent modification for peptide sequencing by postsource decay-matrix assisted laser desorption/ionization-mass spectrometry. *Electrophoresis* **21:** (9) 1651.

Nyman TA, Matikainen S, Sareneva T, Julkunen I, Kalkkinen N. 2000. Biocity, Turku Ctr Biotechnol, POB 123, FI-20521 Turku, Finland. Proteome analysis reveals ubiquitin-conjugating enzymes to be a new family of interferon-α-regulated genes. *Eur J Biochem* **267:** (13) 4011.

Plowman JE, Bryson WG, Jordan TW. 2000. Wool Res Org New Zealand, Private Bag 4749, Christchurch, New Zealand. Application of proteomics for determining protein markers for wool quality traits. *Electrophoresis* **21:** (9) 1899.

Copyright © 2001 John Wiley & Sons, Ltd. 

Seow TK, Ong SE, Liang RCMY, Ren EC, Chan L, Ou K, Chung MCM*. 2000. *Natl Univ Singapore, Clin Res Ctr, Bioproc Technol Ctr, SG-119260 Singapore, Rep Singapore. Two-dimensional electrophoresis map of the human hepatocellular carcinoma cell line, HCC-M, and identification of the separated proteins by mass spectrometry. *Electrophoresis* **21:** (9) 1787.

Simpson RJ, Connolly LM, Eddes JS, Pereira JJ, Moritz RL, Reid GE. 2000. Royal Melbourne Hosp, Ludwig Inst Canc Res, POB 2008, Parkville, Vic 3050, Australia. Proteomic analysis of the human colon carcinoma cell line (LIM 1215): Development of a membrane protein database. *Electrophoresis* **21:** (9) 1707.

Spahr CS, Susin SA, Bures EJ, Robinson JH, Davis MT, McGinley MD, Kroemer G, Patterson SD*. 2000. *Amgen Inc, 1 Amgen Ctr Dr, Thousand Oaks, Ca 91320, USA. Simplification of complex peptide mixtures for proteomic analysis: Reversible biotinylation of cysteinyl peptides. *Electrophoresis* **21:** (9) 1635.

Takayama M, Tsugita A*. 2000. *Proteomics Res Lab, Amakubo 1-16-1, Tsukuba, Ibaraki 305 0005, Japan. Sequence information of peptides and proteins with in-source decay in matrix assisted laser desorption/ionization-time-of-flight-mass spectrometry. *Electrophoresis* **21:** (9) 1670.

Taoka M, Wakamiya A, Nakayama H, Isobe T. 2000. Tokyo Metropolitan Univ, Grad Sch Sci, Dept Chem, Biochem Lab, Minamiosawa 1-1, Hachioji, Tokyo 192 0397, Japan. Protein profiling of rat cerebella during development. *Electrophoresis* **21:** (9) 1872.

Toda T, Sugimoto M, Omori A, Matsuzaki T, Furuichi Y, Kimura N. 2000. Tokyo Metropolitan Inst Gerontol, Dept Gene Regulat & Prot Funct, Itabashi ku, 35-2 Sakaecho, Tokyo 173, Japan. Proteomic analysis of Epstein-Barr virus-transformed human B-lymphoblastoid cell lines before and after immortalization. *Electrophoresis* **21:** (9) 1814.

Tsugita A, Kawakami T, Uchida T, Sakai T, Kamo M, Matsui T, Watanabe Y, Morimasa T, Hosokawa K, Toda T. 2000. Proteomics Res Lab, 1-16-1 Amakubo, Tsukuba, Ibaraki 305 0005, Japan. Proteome analysis of mouse brain: Two-dimensional electrophoresis profiles of tissue proteins during the course of aging. *Electrophoresis* **21:** (9) 1853.

Ueno I, Sakai T, Yamaoka M, Yoshida R, Tsugita A*. 2000. *Address as above. Analysis of blood plasma proteins in patients with Alzheimer's disease by two-dimensional electrophoresis, sequence homology and immunodetection. *Electrophoresis* **21:** (9) 1832.

Walikonis RS, Jensen ON, Mann M, Provance DW, Mercer JA, Kennedy MB*. 2000. *Calif Technol Inst, Div Biol, Pasadena, Ca 91125, USA. Identification of proteins in the postsynaptic density fraction by mass spectrometry. *J Neurosci* **20:** (11) 4069.

Wang YC, Sun J, Chitnis PR*. 2000. *Iowa State Univ, Dept Biochem Biophys & Mol Biol, 4156 Mol Biol Bldg, Ames, Ia 50011, USA. Proteomic study of the peripheral proteins from thylakoid membranes of the cyanobacterium *Synechocystis* sp PCC 6803. *Electrophoresis* **21:** (9) 1746.

Yanagida M, Miura Y, Yagasaki K, Taoka M, Isobe T, Takahashi N. 2000. Tokyo Univ Agr & Technol, 3-5-8 Saiwan cho, Fuchu, Tokyo 183 8509, Japan. Matrix assisted laser desorption/ionization-time of flight-mass spectrometry analysis of proteins detected by anti-phosphotyrosine antibody on two-dimensional-gels of fibroblast cell lysates after tumor necrosis factor-α stimulation. *Electrophoresis* **21:** (9) 1890.

### 12 Protein structural genomics

Domingues FS, Koppensteiner WA, Sippl MJ*. 2000. *Salzburg Univ, Inst Chem & Biochem, Ctr Appl Mol Engn, Jakob Haringer Str 3, AU-5020 Salzburg, Austria. The role of protein structure in genomics. *FEBS Lett* **476:** (1-2) 98.

Grishin NV, Wolf YI, Koonin EV. 2000. Univ Texas, SW Med Ctr, 5323 Harry Hines Blvd, Dallas, Tx 75235, USA. From complete genomes to measures of substitution rate variability within and between proteins. *Genome Res* **10:** (7) 991.

Kelley LA, MacCallum RM, Sternberg MJE*. 2000. *Imperial Canc Res Fund, Biomolec Modelling Lab, 44 Lincolns Inn Fields, London WC2A 3PX, England. Enhanced genome annotation using structural profiles in the program 3D-PSSM. *J Mol Biol* **299:** (2) 499.

Murayama K, Shindo N, Suzuki R, Kawakami M, Mineki R, Taka H, Kazuno S, Nagata K, Maruyama T, Tanokura M. 2000. Juntendo Univ, Sch Med, Cent Lab Med Sci, Div Biochem Anal, Bunkyo ku, 2-1-1 Hongo, Tokyo 113 8421, Japan. Characterization of native and recombinant peptidyl propyl *cis-trans* isomerases derived from *Methanococcus thermolithotrophicus* based on cDNA sequence. *Electrophoresis* **21:** (9) 1733.

Schwede T, Diemand A, Guex N, Peitsch MC*. 2000. Swiss Inst Bioinformat, Glaxo Wellcome Expt Res, 16 Chemin Aulx, CH-1228 Plan-les-Ouates, Switzerland. Protein structure computing in the genomic era. *Res Microbiol* **151:** (2) 107.

Wolf YI, Grishin NV*, Koonin EV. 2000. *Univ Texas, SW Med Ctr, Dept Biochem, 5323 Harry Hines Blvd, Dallas, Tx 75390, USA. Estimating the number of protein folds and families from complete genome data. *J Mol Biol* **299:** (4) 897.

### 14 Genomic approaches to development

Abdelilah-Seyfried S, Chan YM, Zeng CY, Justice NJ, Younger-Shepherd S, Sharp LE, Barbel S, Meadows SA, Jan LY, Jan YN*. 2000. *Univ Calif, Howard Hughes Med Inst, Dept Physiol, 3rd & Parnassus Ave, San Francisco, Ca 94143, USA. A gain-of-function screen for genes that affect the development of the *Drosophila* adult external sensory organ. *Genetics* **155:** (2) 733.

Abidari JM, Gonzales ET, Inoue K, Lupski JR, Karsenty G, Katsanis N*. 2000. *Baylor Coll Med, Dept Mol & Human Genet, Room 610E, Houston, Tx 77030, USA. Identification of novel genes expressed during metanephric induction through single-cell library screening. *Kidney Int* **57:** (6) 2221.

Berns K, Hijmans EM, Koh E, Daley GQ, Bernards R*. 2000. *Netherlands Canc Inst, Div Mol Carcinogenesis, Plesmanlaan 121, NL-1066 CX Amsterdam, The Netherlands. A genetic screen to identify genes that rescue the slow growth phenotype of c-*myc* null fibroblasts. *Oncogene* **19:** (29) 3330.

Ferrandiz C, Liljegren SJ, Yanofsky MF*. 2000. *Univ Calif San Diego, Sect Cell & Dev Biol, La Jolla, Ca 92093, USA. Negative regulation of the *SHATTERPROOF* genes by *FRUITFULL* during *Arabidopsis* fruit development. *Science* **289:** (5478) 436.

Mendoza L, Alvarez-Buylla ER*. 2000. *Univ Nacl Autonoma Mexico, Inst Ecol, Lab Genet Mol & Evolucion, AP Postal 70-275, MX-04510 Mexico City, DF, Mexico. Genetic regulation of root hair development in *Arabidopsis thaliana*: A network model. *J Theor Biol* **204:** (3) 311.

Yoshioka K, Matsuda F, Takakura K, Noda Y, Imakawa K*, Sakai S. 2000. *Univ Tokyo, Fac Agr, Lab Anim Breeding, Bunkyo ku, 1-1-1 Yayoi, Tokyo 113 8657, Japan. Determination of genes involved in the process of implantation: Application of GeneChip to scan 6500 genes. *Biochem Biophys Res Commun* **272:** (2) 531.

### 15 Technological advances

Alyapkina YS, Romanova YM, Alekseeva NV, Kovalev YN, Gaintseva AV, Gintsburg AL. 2000. Russian Acad Med Sci, NF Gamaleya Epidemiol & Microbiol Res Inst, RU-123098 Moscow, Russia. Development of a quantitative PCR technique and its application to the evaluation of gene expression. *Russ J Genet* **36:** (7) 821.

Brenner S, Johnson M, Bridgham J, Golda G, Lloyd DH, Johnson D, Luo SJ, McCurdy S, Foy M, Ewan M, Roth R, George D, Eletr S, Albrecht G, Vermaas E, Williams SR, Moon K, Burcham T, Pallas M, DuBridge RB, Kirchner J, Fearon K, Mao J, Corcoran K. 2000. Lynx

Copyright © 2001 John Wiley & Sons, Ltd. 

Therapeut Inc, 25861 Ind Blvd, Hayward, Ca 94545, USA. Gene expression analysis by massively parallel signature sequencing (MPSS) on microbead arrays. *Nat Biotechnol* **18:** (6) 630.

Haddad R, Morrow AD, Plass C, Held WA*. 2000. *Roswell Park Canc Inst, Dept Mol & Cellular Biol, Buffalo, NY 14263, USA. Restriction Landmark Genomic Scanning of mouse liver tumors for gene amplification: Overexpression of cyclin A2. *Biochem Biophys Res Commun* **274:** (1) 188.

Huang JC, Sun M*. 2000. *Univ Hong Kong, Dept Zool, Pokfulam Rd, Hong Kong, Peoples Rep China. Fluorescein PAGE analysis of microsatellite-primed PCR: A fast and efficient approach for genomic fingerprinting. *Biotechniques* **28:** (6) 1068.

Lehmann U, Glockner S, Kleeberger W, Feist H, Von Wasielewski R, Kreipe H. 2000. Med Sch, Inst Pathol, Carl Neuberg Str 1, DE-30625 Hannover, Germany. Detection of gene amplification in archival breast cancer specimens by laser-assisted microdissection and quantitative real-time polymerase chain reaction. *Am J Pathol* **156:** (6) 1855.

McGinley MD, Davis MT, Robinson JH, Spahr CS, Bures EJ, Beierle J, Mort J, Patterson SD*. 2000. *Amgen Inc, Biochem, 1 Amgen Ctr Dr, Thousand Oaks, Ca 91320, USA. A simplified device for protein identification by microcapillary gradient liquid chromatography-tandem mass spectrometry. *Electrophoresis* **21:** (9) 1678.

Ohishi M, Satoh M, Maeda T. 2000. Kitasato Univ, Sch Sci, Dept Phys, Lab Biomol Dynamics, 1-15-1 Kitasato, Sagamihara, Kanagawa 228 855, Japan. Preparative two-dimensional gel electrophoresis with agarose gels in the first dimension for high molecular mass proteins. *Electrophoresis* **21:** (9) 1653.

Rondon MR, August PR, Bettermann AD, Brady SF, Grossman TH, Liles MR, Loiacono KA, Lynch BA, MacNeil IA, Minor C, Tiong CL, Gilman M, Osburne MS, Clardy J, Handelsman J*, Goodman RM. 2000. *Univ Wisconsin, Dept Plant Pathol, 1630 Linden Dr, Madison, Wi 53706, USA. Cloning the soil metagenome: A strategy for accessing the genetic and functional diversity of uncultured microorganisms. *Appl Environ Microbiol* **66:** (6) 2541.

Su XZ, Carlton JMR. 2000. NIH/NIAID, Parasit Dis Lab, Room 126, Bldg 4, 4 Ctr Dr MSC 0425, Bethesda, Md 20892, USA. Genome display and typing of *Plasmodium* parasites using anchored polyA and polyT oligonucleotides. *Exp Parasitol* **94:** (4) 273.

Talaat AM, Hunter P, Johnston SA*. 2000. *Univ Texas, SW Med Ctr, Ctr Biomed Invent, 5323 Harry Hines Blvd, Dallas, Tx 75390, USA. Genome-directed primers for selective labeling of bacterial transcripts for DNA microarray analysis. *Nat Biotechnol* **18:** (6) 679.

### 16 Bioinformatics

Baldi P. 2000. Univ Calif, Dept Informat & Comp Sci, Irvine, Ca 92697, USA. On the convergence of a clustering algorithm for protein coding regions in microbial genomes. *Bioinformatics* **16:** (4) 367.

Fernandez-de Cossio J, Gonzalez J, Satomi T, Shima T, Okumura N, Besada V, Betancourt L, Padron G, Shimonishi Y, Takao T*. 2000. *Osaka Univ, Inst Prot Res, Div Organ Chem, Yamadaoka 3-2, Suita, Osaka 565 0871, Japan. Automated interpretation of low-energy collision-induced dissociation spectra by SeqMS, a software aid for *de novo* sequencing by tandem mass spectrometry. *Electrophoresis* **21:** (9) 1694.

Glusman G, Lancet DO. 2000. Weizmann Inst Sci, Dept Mol Genet, IL-76100 Rehovot, Israel. GESTALT: A workbench for automatic integration and visualization of large-scale genomic sequence analyses. *Bioinformatics* **16:** (5) 482.

Gotoh O. 2000. Saitama Canc Ctr, Res Inst, 818 Komuro, Ina, Saitama 362 0806, Japan. Homology-based gene structure prediction: Simplified matching algorithm using a translated codon (tron) and improved accuracy by allowing for long gaps. *Bioinformatics* **16:** (3) 190.

Halushka MK, Mathews DJ, Bailey JA, Chakravarti A*. 1999. *Case Western Reserve Univ, Sch Med, Dept Genet, BRB 721, 10900 Euclid Ave, Cleveland, Oh 44106, USA. GIST: A web tool for collecting gene information. *Physiol Genomics* **1:** (2) 75.

Henikoff JG, Pietrokovski S, McCallum CM, Henikoff S*. 2000. *Howard Hughes Med Inst, Fred Hutchinson Canc Res Ctr, 1100 Fairview Ave Nth, POB 19024, Seattle, Wa 98109, USA. Blocks-based methods for detecting protein homology. *Electrophoresis* **21:** (9) 1700.

Hiscock D, Upton C*. 2000. *Univ Victoria, Dept Biochem & Microbiol, POB 3055, Victoria, Brit Columbia, Canada V8W 3P6. Viral Genome DataBase: Storing and analyzing genes and proteins from complete viral genomes. *Bioinformatics* **16:** (5) 484.

Hu YJ, Sandmeyer S, McLaughlin C, Kibler D. 2000. Tatung Univ, Taipei, Taiwan. Combinatorial motif analysis and hypothesis generation on a genomic scale. *Bioinformatics* **16:** (3) 222.

Jensen LJ, Knudsen S. 2000. Tech Univ Denmark, Dept Biotechnol, Ctr Biol Sequence Anal, Bldg 208, DK-2800 Lyngby, Denmark. Automatic discovery of regulatory patterns in promoter regions based on whole cell expression data and functional annotation. *Bioinformatics* **16:** (4) 326.

Lang DM. 2000. 2538 Great Highway, San Francisco, Ca 94116, USA. Net Nearest Neighbor Analysis (NNNA) summarizes non-compensated dinucleotides within gene sequences. *Bioinformatics* **16:** (3) 212.

Lash AE, Tolstoshev CM, Wagner L, Schuler GD, Strausberg RL, Riggins GJ, Altschul SF. 2000. NIH/Natl Lib Med, Natl Ctr Biotechnol Informat, Bethesda, Md 20894, USA. SAGEmap: A public gene expression resource. *Genome Res* **10:** (7) 1051.

Massingham T, Goldman N. 2000. Univ Cambridge, Dept Zool, Museum Zool, Downing St, Cambridge CB2 3EJ, England. EDIBLE: Experimental Design and Information calculations in phylogenetics. *Bioinformatics* **16:** (3) 294.

McGuire AM, Hughes JD, Church GM*. 2000. *Harvard Univ, Sch Med, Lipper Ctr Computat Genet, Grad Program Biophys, Boston, Ma 02115, USA. Conservation of DNA regulatory motifs and discovery of new motifs in microbial genomes. *Genome Res* **10:** (6) 744.

Parsons JD, Rodriguez-Tome P. 2000. Cereon Genomics, 45 Sidney St, Cambridge, Ma 02139, USA. JESAM: CORBA software components to create and publish EST alignments and clusters. *Bioinformatics* **16:** (4) 313.

Pocock MR, Hubbard TJP. 2000. Sanger Ctr, Wellcome Trust Genome Campus, Cambridge CB10 1SA, England. A browser for expression data. *Bioinformatics* **16:** (4) 402.

Rambaut A. 2000. Univ Oxford, Dept Zool, Sth Parks Rd, Oxford OX1 3PS, England. Estimating the rate of molecular evolution: Incorporating non-contemporaneous sequences into maximum likelihood phylogenies. *Bioinformatics* **16:** (4) 395.

Robinson-Rechavi M, Huchon D. 2000. Ecole Normale Super Lyon, Lab Biol Mol & Cellulaire, 6 Allee Italie, FR-69364 Lyon 07, France. RRTree: Relative-Rate Tests between groups of sequences on a phylogenetic tree. *Bioinformatics* **16:** (3) 296.

Strehlow D. 2000. Boston Univ, Dept Med, Med Ctr, 80 East Concord St, Boston, Ma 02118, USA. Software for quantitation and visualization of expression array data. *Biotechniques* **29:** (1) 118.

Streicher J, Donat MA, Strauss B, Sporle R, Schughart K, Muller GB. 2000. Univ Vienna, Dept Anat, Integrat Morphol Grp, Vienna, Austria. Computer-based three-dimensional visualization of developmental gene expression. *Nat Genet* **25:** (2) 147.

Usuka J, Zhu W, Brendel V*. 2000. *Iowa State Univ, Dept Zool & Genet, 2112 Mol Biol Bldg, Ames, Ia 50011, USA. Optimal spliced alignment of homologous cDNA to a genomic DNA template. *Bioinformatics* **16:** (3) 203.

Wooding S. 2000. Univ Utah, Dept Anthropol, 270 Sth 1400 East, Salt Lake City, Ut 84112, USA. PRoMT: Inferring demographic history from DNA sequences. *Bioinformatics* **16:** (3) 298.

Xie GC, De Marco R, Blevins R, Wang YH. 2000. Merck & Co Inc, Dept Bioinformat, West Point, Pa 19486, USA. Storing biological sequence databases in relational form. *Bioinformatics* **16:** (3) 288.

Copyright © 2001 John Wiley & Sons, Ltd. 
